# Meeting Abstracts from the 67^th^ Annual British Thyroid Association Meeting

**DOI:** 10.1186/s13044-019-0069-x

**Published:** 2019-11-19

## L1 George Murray Lecture

### Graves’ Hyperthyroidism and Orbitopathy - Guidelines and Novel Treatment Strategies

#### George Kahaly (gkahaly@uni-mainz.de)

##### Johannes Gutenberg University Medical Center, Mainz, Germany

Graves’ disease (GD) is an inflammatory autoimmune condition which is characterised by thyrotropin receptor auto-antibodies (TSHR-Ab). The frequently associated Graves’ orbitopathy (GO) causes substantial morbidity and can result in orbital disfigurement, double vision and visual loss. GO has a substantial negative effect on quality of life, mental health, and socioeconomic status. Smoking, TSHR-Ab titre and duration of thyroid dysfunction are the key risk factors for developing GO in GD. The pathophysiology of GD and especially GO has been revised with identification of new potential therapeutic targets. Recent clinical trials have shown that considerable benefit may be derived from the addition of anti-proliferative agents, e.g. mycophenolate sodium in preventing deterioration after steroid cessation. In addition, targeted biologic therapies have shown promise, including teprotumumab (anti-IGF-1R monoclonal antibody) which substantially reduces proptosis, rituximab (anti-CD20) which reduces inflammation and tocilizumab (anti-IL6-R) which potentially benefits both of these parameters. This lecture therefore outlines the optimal management of GD and GO and summarises the recent research developments in this area.

## S1 Pathogenesis of alemtuzumab-induced thyroid autoimmunity

### Joanne Jones (jls53@medschl.cam.ac.uk)

#### University of Cambridge & Addenbrooke’s Hospital, Cambridge, UK

The talk will cover our current understanding as to why nearly 50% of individuals with relapsing remitting multiple sclerosis develop thyroid autoimmunity (primarily Graves’ disease) as their immune system reconstitutes following treatment with the lymphocyte depleting humanised anti-CD52 monoclonal antibody alemtuzumab (Lemtrada).

## S2 Key advances in studies of the TSHR and controlling TSHR activity

### Paul Sanders (firs@rsrltd.eclipse.co.uk)

#### FIRS Laboratories, RSR Ltd, Parc Ty Glas, Llanishen, Cardiff, CF14 5DU, UK

Advances in studies of the TSHR and controlling TSHR activity are described. The crystal structures of the thyroid stimulating hormone receptor (TSHR) leucine rich domain (amino acids 22-260) were solved in complex with a stimulating human monoclonal autoantibody (M22) and a blocking human autoantibody (K1-70). However, attempts to purify and crystallise ligand-free TSHR260 have been unsuccessful due to poor stability. Stable TSHR260-JMG55 was produced by mutagenesis, expressed in insect cells and purified using ion exchange chromatography, affinity chromatography, nickel-affinity chromatography and size-exclusion chromatography. Purified ligand-free TSHR260-JMG55 was deglycosylated and crystallised, and the structure solved to 2.83Å resolution. Ligand free TSHR260-JMG55 was approximately 900 times more thermostable than wild type TSHR, and bound TSHR monoclonal autoantibodies and patient serum autoantibodies with similar affinity to wild-type TSHR260. Stimulation of cyclic AMP was comparable in CHO cells transfected with full length wild-type TSHR and full length TSHR- JMG-55. Crystal structure analysis of TSHR260-JMG55 demonstrated remarkable similarity to the TSHR260 bound to M22 or K1-70. Thermostable TSHR260-JMG55 should be useful in designing new methods for TSHR autoantibody detection and in developing new strategies for treating TSHR autoimmunity.

The human monoclonal blocking TSHR autoantibody K1-70 offers a potential strategy for controlling TSHR activity in Graves’ disease (GD) and Graves’ ophthalmopathy (GO) and to also block TSHR signalling in advanced, well differentiated thyroid cancers. K1-70 inhibits cyclic AMP mediated TSHR signalling by TSH or stimulating TSHR autoantibodies (TRAb). K1-70 was administered as an expanded use therapy to a single patient with advanced, well differentiated follicular thyroid carcinoma (FTC), high levels of stimulating TRAb and severe GO. During K1-70 administration (in combination with lenvatinib therapy), thyroid stimulating autoantibody activity decreased from an index of 11 to <1.0, Clinical Activity Score (CAS) improved from 6/7 to 0/7 and exophthalmometry improved from 21mm to 19mm bilaterally. Observations from this study indicate that blocking TSHR activity with K1-70 can be an effective strategy to control GO. Also, there was some evidence that K1-70 had a suppressive effect on the patient’s tumour progression.

## S3 Optimising remission following medical treatment of Graves’ disease

### Prakash Abraham (p.abraham@nhs.net)

#### Aberdeen Royal Infirmary, Aberdeen, UK

The relapse rates of hyperthyroidism following a course of anti- thyroid drugs (ATDs) remains disappointingly high at between 50-70%. Predictors of relapse have been looked at with variable success. These include a recent systematic analysis and predictive models such as the Graves’ Recurrent Events after Therapy (GREAT) score. The major factor influencing relapse is the titre of Thyrotropin Receptor Antibodies. There is potential to identify likely relapse rates of over 80% where perhaps the patient is better served by choosing a definitive treatment option such as radioiodine (RAI) or surgery at an earlier stage. Use of ATDs in early pregnancy is associated with increased risk of congenital anomalies; early ablative treatment (RAI/surgery) should be considered in women of childbearing age at higher risk of relapse of GD.

## S4 Improving survival and cardiovascular outcomes in Graves’ disease

### Onyebuchi Okosieme (OkosiemeOE@cardiff.ac.uk)

#### Thyroid Research Group, Cardiff University School of Medicine, Cardiff CF14 4XN

Hyperthyroidism carries an increased mortality risk. Three well-established treatments for Graves’ disease, namely antithyroid drugs, radioiodine, and surgery, have been available for over 70 years but treatment choices remain highly variable, largely dictated by regional traditions. Yet the impact of disparate treatment approaches on long-term survival have so far remained uncertain. Recent population-based studies using national registries and large patient datasets are now providing fresh insights into modifiable mortality and cardiovascular disease risk factors in the treatment of hyperthyroidism. This presentation will review relevant studies and in particular highlight recently published data from Wales which has shown survival benefits of early and effective control of hyperthyroidism regardless of therapy modality in patients with Graves’ disease. A unified interpretation of existing data and potential avenues for evidence-based change in practice are considered.

## S5 Clinical Update

### Kristien Boelaert (Kristien.Boelaert@uhb.nhs.uk)

#### University of Birmingham & University Hospitals Birmingham, UK

This session will provide an overview of the latest studies in clinical thyroidology. Findings related to cardiovascular outcomes in hyperthyroidism, quality of life in patients in T3/T4 combination therapy, active surveillance of low risk thyroid cancer and thyroid autoimmunity in pregnancy will be presented.

## S6 A year in…basic thyroid hormone research

### Nadia Schoenmakers (naaa2@hermes.cam.ac.uk)

#### University of Cambridge, Metabolic Research Laboratories, Level 4, Wellcome Trust-MRC Institute of Metabolic Science, Box 289, Addenbrooke's Hospital, Cambridge, CB2 0QQ

Dr Schoenmakers will present highlights from research in the field of basic thyroidology.

## O1 Electronic alerts for optimising thyroid hormone replacement in primary care: challenges in pregnancy and the preconception period

### Anh Tran^1,2^, Steve Hyer^3^, Julia Priestley^4^, Onyebuchi Okosieme^5^

#### ^1^Shadbolt Park House Surgery, Worcester Park, UK; ^2^The Longcroft Clinic, Banstead, UK; ^3^Department of Endocrinology, St Helier Hospital, Epsom and St Helier University NHS Trust, Carshalton, UK; ^4^British Thyroid Foundation, UK; ^5^Prince Charles hospital, Cwm Taf Health Board, Merthyr Tydfil, UK

##### **Correspondence:** Anh Tran (dradtran@ok2life.com)

**Introduction:** Suboptimal thyroid function in pregnancy carries significant risks of poor obstetric outcomes including pregnancy loss and neuro-developmental impairment in the offspring. Current UK guidelines recommend TSH target <2.5mU/L at conception and in the first trimester of pregnancy. We recently developed electronic alerts to prompt General Practitioners to test thyroid function and adjust Levothyroxine dose according to current guidelines in patients with primary hypothyroidism.

**Aim:** Our aim in this preliminary audit was to assess the adequacy of thyroid hormone replacement in women of reproductive age with treated primary hypothyroidism, and to evaluate the effects of pregnancy specific electronic alerts on thyroid hormone replacement in this sub-population.

**Methods:** Nine UK practices participated in the study. The study population comprised women aged 15-55 years with treated primary hypothyroidism who were potentially reproductive (not coded with menopause, hysterectomy or sterilization). An electronic protocol which alerts if TSH is out of range, with specific pregnancy alerts when TSH>3mU/L, was developed in EMIS web. Five practices (total population 74,665) had the alerts installed and four (total population 44,166) did not. After 12 months, all nine practices were audited using EMIS Population Reporting Manager. We analysed the percentage of patients with latest TSH above the upper limit of local normal reference range (ULNR) and the percentage of those coded as pregnant or trying to conceive with latest TSH >3mU/L.

**Results:** The prevalence of treated primary hypothyroidism (total hypothyroid population) was 3.02% (n2252) and 2.9% (n1278) in practices with and without alerts respectively. 651 women (2.9% of total hypothyroid population) and 436 women (3.4% of total hypothyroid population) were identified as potentially reproductive, and 35 and 28 were coded as pregnant or trying to conceive in practices with and without alerts respectively. 11% and 15% of the study population, and 9% and 12% of the total hypothyroid population, had latest TSH ULNR in practices with and without alerts respectively. 13/35 (37%) and 8/28 (29%) of women coded as pregnant or trying to conceive had latest TSH > 3mU/L in practices with and without alerts respectively.

**Conclusions:** Between 11-15% of women of reproductive age on thyroid hormone treatment in primary care had suboptimal replacement, and about a third of those coded as pregnant or planning to conceive had TSH >3mU/l. Although an electronic protocol improved adequacy of thyroid hormone replacement in the general population with hypothyroidism, this benefit was not seen in preconception or pregnant women for whom a more stringent TSH target is recommended, although the numbers were small. Further studies looking at strategies to improve TSH optimization in pregnancy and the preconception period are needed.

## O2 Predictors of sub-optimal thyroid hormone replacement in pregnant women with hypothyroidism

### Emily Williams, Kalyani Nagarajah, Onyebuchi Okosieme

#### Diabetes Department, Prince Charles Hospital, Cwm Taf Morgannwg University Health Board, Merthyr Tydfil, Wales, United Kingdom

##### **Correspondence:** Onyebuchi Okosieme (OkosiemeOE@Cardiff.ac.uk)

**Background:** Hypothyroidism affects 2-5% of pregnant women and carries an increased risk of adverse outcomes including pregnancy loss. International guidelines for women with hypothyroidism recommend a TSH concentration of <2.5 mU/L in pregnancy and the preconception period but the feasibility of these targets in real-world clinical practice is unclear.

**Aim:** To determine the prevalence and predictors of sub-optimal thyroid hormone replacement in pregnancy in women with pre-existing hypothyroidism referred to our secondary care specialist antenatal clinic.

**Methods:** We audited clinical and biochemical records of 172 levothyroxine-treated women, mean age 29.9 years, standard deviation 5.5, range 19–43 years. We determined the prevalence of sub-optimal TSH (>2.5 mU/L), and examined factors associated with sub-optimal treatment including preconception TSH and levothyroxine dose requirements.

**Results:** Median gestational age at first thyroid function test was 12 weeks, interquartile range 8-16 weeks. Preconception TSH in the 12-months preceding pregnancy was >2.5 mU/L in 49% of women, of which 77% remained >2.5 mU/L in pregnancy. Post-conception TSH was >2.5 mU/L, >4.0 mU/L, and >10.0 mU/L in 59%, 37% and 7% of women respectively. Sub-optimal TSH in pregnancy was not associated with age, parity, disease aetiology, levothyroxine requirements or dose adjustments. In multivariable logistic regression, only a preconception TSH >2.5 mU/L predicted sub-optimal gestational TSH (p 0.002).

**Conclusion:** A significant proportion of levothyroxine-treated women fail to achieve international treatment targets during pregnancy. Women at risk of sub-optimal thyroid hormone replacement in pregnancy are identifiable before conception and a systematic pre-conception approach is needed to improve adherence to guideline targets.

## O3 Thyroid hormone withdrawal prior to radioiodine therapy for differentiated thyroid cancer – Impact on renal function and I^131^ clearance

### Mohammad Sadiq J^1^, Alex J Graveling^1^, Fergus McKiddie^2^, Rebecca Duguid^3^, Prakash Abraham^1^

#### ^1^Department of Endocrinology, Aberdeen Royal Infirmary, NHS Grampian; ^2^Nuclear Medicine, Aberdeen Royal Infirmary, NHS Grampian; ^3^Radiation Protection, Aberdeen Royal Infirmary, NHS Grampian

##### **Correspondence:** Mohammad Sadiq J (mohammad.jeeyavudeen@nhs.net)

**Background:** Thyroid hormone withdrawal (THW) is the traditional method of preparation for radioiodine therapy for differentiated thyroid cancer. Studies have shown that THW may impair renal function and delay radioiodine clearance. This study examined renal function during THW and its influence on I^131^ clearance.

**Methods:** Fifty-seven patients who received high dose I^131^ therapy (1,148-5,760 MBq) preceded by THW were retrospectively analysed. Baseline thyroid and renal function in the euthyroid pre-operative and hypothyroid (TSH >30mU/L) state were obtained along with the radiation dose administrated and dose emission rate at 1 metre prior to discharge (day 4 or 5). GFR was estimated from creatinine values by using the EPI equation.

**Results:** A total of 75 radioiodine treatment episodes were included (13 patients received 2 or more doses) in the analysis. All patients had baseline creatinine within normal range (median 69 and IQR 58-81). There was a significant reduction in GFR from baseline (mean 96.27mls/min, SD 18.41) when compared with GFR in hypothyroid state (mean 74.11mls/min, SD 19.87); t(57) 13.75, p<0.000. This difference remained significant even after a sensitivity analysis of potential confounding variables. Other parameters contributing to the dose rate at discharge were examined (e.g. stimulated pre-treatment thyroglobulin, body mass index).

**Conclusion:** The short period of hypothyroidism experienced by patients undergoing THW prior to radioiodine therapy has a significant impact on renal function. This has shown to influence I^131^ clearance, radiation retention, prolonged hospital stay and radiation protective isolation. A future study will examine whether using recombinant TSH injections, rather than THW, ameliorates the decline in renal function and reduces hospital stay.

## O4 VCP: A novel interactor of the sodium iodide symporter, which can be pharmacologically targeted to increase radioiodine uptake in human and mouse thyrocytes

### Alice Fletcher, Martin L. Read, Caitlin E. M. Thornton, Vikki L. Poole, Kristien Boelaert, Vicki E. Smith±, Christopher J. McCabe±

#### Institute of Metabolism and Systems Research, University of Birmingham, Birmingham, United Kingdom, B15 2TT, UK

##### **Correspondence:** Christopher J. McCabe (mccabcjz@bham.ac.uk)

±joint senior authors

By exploiting the sodium iodide symporter (NIS), ablative radioiodine therapy is an effective treatment for papillary thyroid cancer (PTC). Unfortunately, >25% of PTC patients are unable to accumulate therapeutically sufficient radioiodine due to NIS dysregulation, and have reduced mean survival times. Radioiodine therapy has been proposed as a viable treatment for breast cancer but is hampered by diminished membranous NIS. Currently, the regulation of NIS localisation remains ill-defined.

Mass spectrometry, co-immunoprecipitation and proximity ligation assays identified and validated VCP as a novel NIS interactor. VCP siRNA-depletion increased radioiodine uptake in lentivirally-expressing-NIS breast and thyroid cancer cells, and significantly boosted endogenous NIS function in human thyrocytes. Conversely, VCP-overexpression repressed radioiodine uptake, accompanied by lowered membranous NIS. Five VCP inhibitors all overcame VCP inhibition of NIS function in our transformed cells. Notably, FDA-approved VCP inhibitors Clotrimazole and Ebastine augmented radioiodine uptake in mouse and human thyrocytes.

TCGA analyses revealed VCP mRNA expression is significantly upregulated in PTC, providing a putative explanation for repressed NIS function. High tumoral VCP expression is associated with a worse disease-free survival when compared to low VCP expression. Strikingly, in patients who receive radioiodine, high tumoral VCP expression results in a markedly worse disease-free survival, indicating high VCP expression correlates with a worse response to radioiodine therapy.

Our data reveal a new pathway of NIS regulation. Critically, FDA-approved VCP inhibitors highlight a novel potential therapeutic strategy for enhancing radioiodine uptake in PTC patients while increasing the feasibility of radioiodine therapy in breast cancer via transient inhibition of VCP activity.

## O5 Novel driver events in thyroid cancer recurrence

### Hannah Nieto^1^, Caitlin Thornton^1^, Alice Fletcher^1^, Albert Nobre de Menezes^2,3^, Katie Brookes^1^, Mohammed Alshahrani^1^, Martin Read^1^, Kristien Boelaert^1^, Vicki Smith^1^, Jean-Baptiste Cazier^2,3^, Hisham Mehanna^4^, Chris McCabe^1^

#### ^1^Institute of Metabolism and Systems Research, University of Birmingham, Birmingham, UK; ^2^Institute of Cancer and Genomic Science, University of Birmingham, Birmingham, UK; ^3^Centre for Computational Biology, University of Birmingham, Birmingham, UK; ^4^Institute of Head and Neck Studies and Education (InHANSE), University of Birmingham, Birmingham, UK

##### **Correspondence:** Hannah Nieto (dixonh@adf.bham.ac.uk)

The incidence of thyroid cancer is increasing worldwide. Whilst outcome in thyroid cancer is generally good, up to 25% of patients develop recurrence, and have a significantly reduced life expectancy. We hypothesise those thyroid tumours which subsequently recur display a distinct pattern of driver events. Whole exome sequencing data were downloaded from The Cancer Genome Atlas (TCGA). Bioinformatic analysis of data on N=43 patients whose tumours recurred was performed, using a Platypus, Annovar and SIFT/PolyPhen2/MutationTaster filtering pipeline. This identified mutations in biologically significant genes, including Inosine-5′-monophosphate dehydrogenase 2 (IMPDH2), 6-Phosphofructo-2-Kinase/Fructose-2,6-Biphosphatase 4 (PFKFB4) and Dicer 1 ribonuclease type III (DICER1). As in-silico analysis suggested these variants to be pathogenic, we recreated these mutations. Subcellular localisation, proliferation, cellular migration and invasion were investigated in cell lines which represent the most common background driver mutations of papillary thyroid cancer (TPC1: RET/PTC; SW1736: BRAF; Cal62: Ras). In TPC1 cells IMPDH2 mutation significantly increased cell migration at 4, 8 and 24hrs vs. WT (p=0.0068, p=0.0008, p=0.0088 respectively) and DICER1 mutation induced increased cell migration at 24 hours vs. vector-only (p=0.0094). Overexpression of IMPDH2 resulted in altered intracellular localisation into intracellular discrete bodies known as rods and rings. As recurrence may also reflect altered gene expression, we analysed the RNA and microRNA profile of the recurrent patients (N=43) compared to the non-recurrent (N=457). In particular, genes involved in matrix adhesion and thyroid cancer pathogenesis were most differentially expressed in recurrence patients, including fibronectin 1 (FN1), thyroglobulin (TG), α3 integrin (ITGA3), SPARC-like protein 1 (SPARCL1), and the proto-oncogene mesenchymal-epithelial transition factor (MET) (p=0.00376, p=0.00311, p=0.00757, p=0.01874, p=0.00003, p=0.00003 respectively). Overall, we propose that rare somatic mutations on top of established driver events, as well as specifically altered RNA expression levels, may be key to predicting thyroid cancer recurrence.

## O6 Identification of *THRA* variants in genome datasets that could cause Resistance to Thyroid Hormone alpha

### Maura Agostini^1^, Beatriz Romartinez^2^, Carla Moran^1^, Odelia Rajanayagam^1^, Greta Lyons^1^, Louise Fairall^2^, John Schwabe^2^, Krishna Chatterjee^1^

#### ^1^Wellcome Trust-MRC Institute of Metabolic Science, University of Cambridge, Cambridge, UK; ^2^Institute of Structural and Chemical Biology, University of Leicester, UK

##### **Correspondence:** Krishna Chatterjee (kkc1@medschl.cam.ac.uk)

Over 150 different *THRB* mutations causing Resistance to Thyroid Hormone (RTH) β, a disorder with a distinct thyroid biochemical phenotype, have been reported. However, only 22 different mutations in the highly homologous *THRA* gene causing RTHα have been recorded, suggesting that this disorder with near-normal thyroid hormone levels is under-ascertained.

Following identification of RTHα via the UK 100K genome project, we searched datasets (ExAc/GnomAD; DiscovEHR) containing 200,000 exomes, identifying 57 rare, non-synonymous, heterozygous variants in the ligand binding domain (LBD) of thyroid hormone receptor α1. 27/57 TRα1 variants were deemed to be potentially pathogenic, either because the aminoacid substitutions involved residues that are known to be mutated in RTHβ or aminoacid changes were predicted to be deleterious when modelled on the TRα1 LBD crystal structure. When tested in functional assays calibrated with TRα1 mutants known to cause RTHα, 7/27 different TRα1 variants were significantly transcriptionally impaired and inhibited wild type receptor action in a dominant negative manner. Using this approach, we are assessing the deleteriousness of 26 rare *THRA* LBD variants, identified in 50,000 exomes from UK Biobank, and correlating their properties with clinical and biochemical phenotypes.

Our identification of potentially pathogenic TRα1 variants in exome datasets at a frequency (~1 in 28,000) comparable to the prevalence (1 in 40,000) of RTHβ, suggests that RTHα is underdiagnosed. Our studies also provide an opportunity to widen the spectrum of phenotypes associated with RTHα and identify subjects in whom thyroxine therapy can be trialed.

## P1 T3 Thyrotoxicosis caused by a struma ovarii characterised by a paradoxical Free T4 rise with carbimazole therapy

### James Prentice^1^, Kate Panter^2^, Ayoma Attygalle^3^, Thomas Ind^3^, Malcolm Prentice^1^

#### ^1^Croydon University Hospital, London, UK; ^2^Guys Hospital, London, UK; ^3^Royal Marsden Hospital London, UK

##### **Correspondence:** Malcolm Prentice (malcolmprentice@nhs.net)

A 33-year-old female presented with 1-year history of intermittent left iliac fossa pain, secondary amenorrhoea, a gradual onset of feeling hot, sweating and weight loss. On examination she was euthyroid with a solitary 1.5 cm right U3 nodule and with an ovarian dermoid enlarged to 11.6cm on ultrasound.

Pre-operative tests showed TSH <0.02 mU/L (0.27-4.2), FT4 5.5 pmol/L (10-28), FT3 7.0 pmol/L (3.1 – 6.8). TSH receptor and thyroid autoantibodies were negative. Technetium scan showed low 0.2% homogeneous uptake. Nodule FNA was reported as Thy2.

Carbimazole 10 mg was started. Ovarian cystectomy was performed on day 17. Pathology showed struma ovarii. On day 23 the FT3 3.6 pmol/L, TSH 11.9 mU/l, and FT4 rose from 3.9 to 7.7 pmol/L, carbimazole was reduced to 5mg and stopped on day 50. The FT3 and TSH remained normal. The FT4 continued to rise to normal 10.9 pmol/L by day 100. This is a rare cause of pure T3 thyrotoxicosis from a struma ovarii.

The patient gave written consent to this publication.

## P2 Malignant struma ovarii: an uncommon presentation of thyroid cancer (case series)

### Mohammad Sadiq J^1^, Alex J. Graveling^1^, Muhammad Shakeel^2^, Louise Smart^3^, Prakash Abraham^1^

#### ^1^Department of Endocrinology, Aberdeen Royal Infirmary; ^2^ENT Department, Aberdeen Royal Infirmary; ^3^Department of Pathology, Aberdeen Royal Infirmary

##### **Correspondence:** Mohammad Sadiq J (mohammad.jeeyavudeen@nhs.net)

**Background**: Ectopic thyroid tissue has been found throughout the body achieving clinical significance from hormonal hypersecretion or malignant transformation. Thyroid carcinoma arising in malignant struma ovarii (MSO) is rare and lacks specific management guidelines. The usual sequence of treatment is staging laparotomy, followed by total thyroidectomy and radioiodine therapy in higher risk cases.

**Case description**: Four patients were diagnosed with MSO in the last five years at a single tertiary hospital (see below table). All reported abdominal pain with a pelvic mass on examination.

**Table:** 

	Case 1	Case 2	Case 3	Case 4
Age at diagnosis (years)	49	45	52	71
TSH (mU/L) at diagnosis	1.62	1.7	2.69	2.63
Tumour size(cm)	10	9.5	9.3	9
Treatment received	BSO*	BSO*+TT^¥^	BSO*+TT^¥^	BSO*+TT^¥^
High dose radioiodine therapy	Nil	3 doses	1 dose	1 dose
Pre-radioiodine thyroglobulin (ug/L)	19	5417	28	12
Post treatment thyroglobulin (ug/L)	15	24	<1	<1
RET (10q11) mutation	-	Negative	Negative	-
Final MSO Histology	Papillary microcarcinoma	FVPTC^ψ^	FVPTC^ψ^	FVPTC^ψ^

^*^Bilateral salpingo-oophorectomy, ¥-Total thyroidectomy, ψ-Follicular variant papillary thyroid cancer

Case 1 had papillary microcarcinoma, rarely seen with MSO. With a low risk of recurrence, she was managed with BSO alone and thyroglobulin remains stable. Case 2 had macronodular lung metastasis and is doing well after repeated radioiodine therapy. Cases 3 and 4 have undetectable thyroglobulin following surgery and radioiodine. Case 4 presented at a later age with the rare finding of coexistent borderline mucinous cystic tumour.

**Discussion:** This is a rare tumour with no specific clinical presentation and standard treatment guidelines pose challenges to the treating team.

## P3

**Abstract withdrawn**

## P4 Hyper or hypo? Factors affecting phenotype in alemtuzumab-induced thyroid autoimmunity

### Moustaki M, Chung TT

#### Department of Diabetes and Endocrinology, University College London Hospital, London, UK

##### **Correspondence:** Chung TT (teng-teng.chung@nhs.net)

**Case series:** We present three cases of alemtuzumab-induced thyroid dysfunction (Table 1), in premenopausal women treated with alemtuzumab for multiple sclerosis (MS). The first two patients presented with overt hyperthyroidism 12 months after their second course of alemtuzumab and were treated with carbimazole. The third case initially presented with subclinical hyperthyroidism 10 months after her first alemtuzumab infusion, followed by profound hypothyroidism within 1 month.

**Clinical characteristics:** All cases had positive anti-TSH receptor antibodies (TRAB; Table 1). Two cases presented with hyperthyroidism and one with hypothyroidism, while shifting between the two was noticed in patients 2 and 3. In particular, patient 2 demonstrated a tri-phasic hyper-hypo-hyper profile. Despite high initial fT4 levels, the first 2 patients had mild thyrotoxic symptoms and achieved biochemical control within 3 months on carbimazole. Likewise, hypothyroidism was asymptomatic in case 3. None of our cases had Graves’ ophthalmopathy.

**Discussion:** Thyroid dysfunction occurs in up to 40% of alemtuzumab-treated patients [1]. MS patients treated with alemtuzumab usually present with Graves’ disease, while interferon-beta treated ones tend to present with Hashimoto’s disease. This is attributed to post-alemtuzumab immune reconstitution syndrome favouring autoantibody-mediated (Thy2/B-cell) processes rather than destructive Thy1-mediated ones [2]. Fluctuations of thyroid activity suggest switching between stimulating and blocking bioactivity of thyroid autoantibodies [1-3]. Hypothyroidism in case 3 could be due to blocking TRAB antibodies or to Hashimoto’s thyroiditis preceded by Hashitoxicosis. Early thyroid atrophy in ultrasound would be specific for the former [3]. Interestingly, TRAB titre was markedly higher during hypothyroid status (Table 1), this is consistent with previous data [1], implying a negative correlation between TRAB titre and bioactivity.

**References**

1. Pariani N, Willis M, Muller I, Healy S, Nasser T, McGowan A, Lyons G, Jones J, et al. Alemtuzumab-induced thyroid dysfunction exhibits distinctive clinical and immunological features. *J Clin Endocrinol Metab*. 2018; 103(8):3010-3018

2. Rotondi M, Molteni M, Leporati P, Capelli V, Marino M, Chiovato L. Autoimmune thyroid diseases in patients treated with alemtuzumab for multiple sclerosis: An example of selective anti-TSH-receptor immune response. Front Endocrinol (Lausanne). 2017:8:254.

3. McLachlan SM, Rapoport B. Thyrotropin-blocking autoantibodies

and thyroid-stimulating autoantibodies: potential mechanisms involved in the pendulum swinging from hypothyroidism to hyperthyroidism or vice versa. *Thyroid*. 2013; 23(1):14-24.

**Table 1 (abstract P4).** Peak value of thyroid function tests and antibodies

	fT4 mmol/L (RR 12-22)		TSH mIU/L (RR 0.27-4.2)		TRAB IU/L (RR 0-1.8)		TPO IU/ml (RR 0-34)		Treatment
	Hyper	Hypo	Hyper	Hypo	Hyper	Hypo	Hyper	Hypo	
Patient 1	64.1	-	<0.01	-	12.6		153		Carbimazole
Patient 2	100	5.0	<0.01	46.8	7.9	84.4	-	-	Carbimazole
Patient 3	22.6	1.9	<0.01	>100		28.2		>600	Levothyroxine

## P5 Macrothyrotropin as a cause of falsely elevated TSH in two clinically euthyroid patients

### Isabelle van Heeswijk^1^, Melanie Griffiths^2^, Steve Jones^3^, Francesca Meakin^2^, Vakkat Muraleedharan^1^

#### ^1^Department of Endocrinology, Kings Mill Hospital, London; ^2^Department of Pathology, Kings Mill Hospital, London; ^3^Department of Haematology, Kings Mill Hospital, London

##### **Correspondence:** Isabelle van Heeswijk (isabelle.vanheeswijk@nhs.net)

**Introduction:** MacroTSH or macrothyrotropin is a complex of TSH with IgG, resulting in a molecule with large molecular mass (>150kDa) but low bioactivity. It can result in falsely elevated TSH whilst T4 levels remain in normal range and the patient is clinically euthyroid, mimicking subclinical hypothyroidism. We describe two cases of clinically euthyroid patients where macroTSH was identified using the polyethylene glycol (PEG) precipitation method.

**Case Description:** The first case concerns a 94 year old gentleman with chronic lymphocytic leukaemia, treated with ibrutinib. A thyroid function test revealed a TSH of 98mU/L. T4 was in the normal range at 14.4pmol/L. A MacroTSH-PEG precipitation study was performed which confirmed the presence of large molecular weight proteins, with a post precipitation TSH of 29mU/L (23% recovery). He was commenced on levothyroxine 25 mcg daily after T4 fell below normal range.

The second case concerns as a 62 year old female whose TFTs were performed due to neuropathy. Initial TSH was 23mU/L and T4 17.9pmol/L. PEG precipitation demonstrated 38% recovery (TSH 8.74mU/L). The patient was not treated due to the corrected TSH <10mU/L and normal T4. Following a course of prednisolone for vasculitis, TSH fell back to normal range.

**Discussion:** The polyethylene glycol precipitation removes high molecular weight proteins that could falsely elevate TSH readings. A low post-PEG TSH recovery indicates the presence of high molecular weight molecules interfering with the assay (including macro-TSH or interfering antibodies). This method was utilised in order to differentiate possible macro-TSH from subclinical hypothyroidism in our patients.

